# Supplementary material for: Functionalized PLGA-Based Nanoparticles with Anti-HSV-2 Human Monoclonal Antibody: A Proof of Concept for Early Diagnosis and Targeted Therapy
Source: Pharmaceutics. 2024 Sep 18;16(9):1218. doi: 10.3390/pharmaceutics16091218 (PMC11434782; doi:10.3390/pharmaceutics16091218)
Supplement: Supplementary file 1 [file pharmaceutics-16-01218-s001.zip › pharmaceutics-3134558-supplementary.pdf]

SUPPORTING INFORMATIONS

FUNCTIONALIZED PLGA-BASED NANOPARTICLES WITH HUMAN MONOCLONAL ANTIBODY: A PROOF OF CONCEPT FOR EARLY DIAGNOSIS AND TARGETED THERAPY

Scientific Reports

Melinda Mariotti [1], Noah Giacon [1], Ettore Lo Cascio [1], Margherita Cacaci [1, 2], Simona Picchietti [3], Maura Di Vito [1], Maurizio Sanguinetti [1, 2], Alessandro Arcovito [1, 4, †, *] and Francesca Bugli [1, 2, †, *]

[†] These authors contributed equally to this work

[*] Correspondence: Alessandro Arcovito alessandro.arcovito@unicatt.it, Francesca Bugli francesca.bugli@unicatt.it

[1] Dipartimento di Scienze Biotecnologiche di Base, Cliniche Intensivologiche e Perioperatorie, Università Cattolica del Sacro Cuore, Largo A. Gemelli 8, 00168 Rome, Italy

[2] Dipartimento di Scienze di Laboratorio e Infettivologiche, Fondazione Policlinico Universitario A. Gemelli IRCCS, 00168 Rome, Italy

[3] Dept. for Innovation in Biological, Agro-food and Forest systems (DIBAF), University of Tuscia, Largo dell'Università snc, 01100 Viterbo, Italy

[4] Fondazione Policlinico Universitario "A. Gemelli", IRCCS, Largo A. Gemelli 8, 00168 Roma, Italy.

S1. METHODS

S1.1 Light and heavy chains cloning

The original plasmid, in which the genes encoding the light and heavy chains had been previously cloned, and the pComb3/TIG vector were digested with XBA I and SAC I restriction enzymes (New England BioLabs, Ipswich, MA, USA), separated on 1% agarose gel, and extracted from the gel using the MinElute Gel Extraction Kit (Qiagen, Hilden, DE) following the protocol described by the manufacturer. The digested LC insert was ligated into the linearized pComb3/TIG vector using T4 DNA Ligase (New England BioLabs, Ipswich, MA, USA). The resulting pComb3/TIG-LC vector was confirmed by restriction analysis.

The original plasmid was used as DNA template for the HC PCR amplification using a modified constant primer $CG1z_{4HIS}$, specifically designed to add a four Histidines tag at the C-terminal end of the HC, and a mix of type A or F

variable primers. Primers used for amplification reactions are reported in Table S1. The KAPA Taq Extra HotStart ReadyMix PCR Kit (KAPABiosystems, Wilmington, MA, USA) containing a blend of KAPA Taq HotStart DNA polymerase and a modified archaeal DNA polymerase possessing proofreading capability was used. The reaction conditions were initial denaturation at 95°C for 2.5 min, followed by 35 cycles of amplification (60 sec at 94°C, 60 sec at 52°C, and 120 sec at 72°C), and a final extension of 7 min at 72°C in the Super Cycler Triple-Zone thermal cycler (Kyratec, Mansfield, QLD, AUS). PCR products were run on agarose gel, stained with ethidium bromide and visualized under ultraviolet light. After purification of the PCR product from the agarose gel, the HC_{HIS} was digested with XHO I and SPE I restriction enzymes and purified from a 1.25% agarose gel. The pComb/TIG-LC vector was digested with corresponding restriction enzymes, and the "Phosphatase, Alkaline from Calf Intestine" (CIP) (Sigma-Aldrich, Saint Louis, MO, USA) was added to remove the 5'-phosphate groups preventing the self-ligation of cleaved DNA vector. After the inactivation of the CIP enzyme, the vector was separated on 1.25% agarose gel and purified from it. The digested HC_{HIS} PCR fragment was inserted into the XHO I-SPE I site of the pComb/TIG-LC vector. A restriction analysis was performed to verify the successful completion of the ligation.

To remove the nucleotide sequence encoding the CP3 protein from the pComb/TIG-LC-HC_{HIS} vector, NHE and SPE I restriction enzymes were used. The digested DNA was separated on 0.85% agarose gel, purified and a self-ligation reaction was performed to close the vector on itself. The pComb3/TIG-LC-HC_{HIS} without CP3 vector was confirmed by restriction analysis.

Table S1. Primers for the heavy chain amplification reactions.

**Primer**	**Nucleotide sequence (5'-3')**
CG1z	GCATGTACTAGTTTTGTCACAAGATTTGGG
$CG1z_{4HIS}$	GCATGTACTAGTCATCACCATCACTTTGTCACAAGATTTGGG
VH1a	CAGGTGCAGCTCGAGCAGTCTGGG
VH3a	GAGGTGCAGCTCGAGGAGTCTGGG
VH6a	CAGGTACAGCTCGAGCAGTCAGG
VH1f	CAGGTGCAGCTGCTCGAGTCTGGG
VH2f	CAGGTGCAGCTACTCGAGTCGGG
VH3f	GAGGTGCAGCTGCTCGAGTCTGGG
VH4f	CAGGTGCAGCTGCTCGAGTCGGG

Table S2. Primers for sequencing the anti-HSV-2 light and heavy chains.

Primer	Nucleotide sequence (5'-3')
SEQKb	ATAGAAGTTGTTCAGCAGGCA
KEF	GAATTCTAAACTAGCTAGTTCG
SEQGb	GTCGTTGACCAGGCAGCCCAG
T3	ATTAACCCTCACTAAAG
CH1	CTGGGCTGCCTGGTCAACGAC

S1.2 Preparation of the Protein G-Human Fab resin

1 mL of Protein G gamma-bind resin (Cytiva, Uppsala, SW) was washed 3 times in PBS, centrifuging each time for 5 min at 3000 rpm. 4 mg of goat anti-human IgG Fab2 fragment secondary antibody (Invitrogen by Thermo Fisher Scientific, Waltham, MA, USA) were added and the resin was incubated for 90 min at room temperature with gentle agitation. The resin was washed two times with 10 Volumes of 0.2 M sodium tetraborate pH 9 (Sigma-Aldrich, Saint Louis, MO, USA), then suspended in sodium tetraborate. Dimethylpimelimidate dihidrochloride (DMP) (5.2 mg/mL) (Sigma-Aldrich, Saint Louis, MO, USA) was used to perform a cross-linking reaction between the Protein G and the antibody and incubated at room temperature for 30 min with gentle agitation. To check the efficiency of the cross-linking, the equivalent of 10 µl of resin was removed before adding the DMP and after its incubation and run in SDS-PAGE in reducing conditions. The resin was washed with 10 volumes of 0.2 M ethanolamine pH 8.0 (Sigma-Aldrich, Saint Louis, MO, USA), then suspended again in 10 volumes of ethanolamine and incubated at room temperature for 2 hours. After four additional washes in PBS, the resin was ready; at its first use, the resin was washed with 10 volumes of elution buffer to remove unbound antibodies and then equilibrated with PBS before the addition of the Fab extract. An aliquot of 1 mL of the lysate was collected as an unpurified sample, and the rest of lysate was applied to the column at a flow rate of 0.5 mL/min to capture the Fab. The column was washed with 30 volumes of PBS; then the Fab was eluted in fractions of 1 mL each using a 100 mM Glycine-HCl pH 2.5 solution as elution buffer, immediately neutralized with 1 M Tris (Sigma-Aldrich, Saint Louis, MO, USA) pH 9. The fractions were analyzed on 12% SDS-PAGE under reducing conditions and stained with the GelCode Blue Safe Protein Stain (Thermo Fisher Scientific, Waltham, MA, USA). Elution fractions with high Fab concentration were pooled and dialyzed against PBS overnight at 4°C. A final SDS-PAGE was performed to make sure that the post-dialysis Fab signal was comparable to the pre-dialysis one. The Fab concentration was determined

using the bicinchoninic acid protein assay (Thermo Fisher Scientific, Waltham, MA, USA) in accordance with the instructions of the manufacturer.

S1.3 SDS-PAGE and Western Blot

Samples were analyzed through sodium dodecyl sulphate-polyacrylamide gel electrophoresis (SDS-PAGE) under reducing conditions. Samples were boiled for 7 min at 99°C before loading onto the gel. The run was performed in 1X Tris/Glycine/SDS Buffer (Bio-Rad Laboratories Inc, Hercules, CA, USA) at 120 V for 90 min. After electrophoresis, the gel was stained with GelCode Blue Safe Protein Stain. The molecular weights of proteins were estimated by comparison with the G252 Opti-protein marker (range of 10-175 kDa) (Applied Biological Materials, Richmond, British Columbia, CA). Proteins in the polyacrylamide gel were transferred onto TransBlot Turbo Mini-size Nitrocellulose membrane using the TransBlot Turbo Transfer System (Bio-Rad Laboratories Inc, Hercules, CA, USA) for 7 min at 25 V. The membrane was incubated with blocking solution (PBS, 5% skim milk powder, and 0.05% Tween20) for 90 min at 37°C, and then incubated with Horseradish peroxidase-labeled goat anti-human IgG (Fab specific) antibody (1:20000) (Sigma-Aldrich, Saint Louis, MO, USA) in PBS, 2% skim milk powder, and 0.05% Tween20. The membrane was washed five times with 0.05% Tween20 in PBS. Signals were detected by chemiluminescence by adding the SuperSignal™ West Dura extended-duration substrate (Thermo Fisher Scientific, Waltham, MA, USA). The result was analyzed using a Bio-Rad ChemiDoc™ imaging system.

Table S3. Amino acid sequences of the Heavy and Light chains of the anti-HSV-2 Fab fragment. The four Histidine tag added to the C-terminal end of the heavy chains constant region is underlined.

	**fr1**	**cdr1**	**fr2**	**cdr2**	**fr3**	**cdr3**	**fr4**
**V_H**	LLESGAEVTKPGASLR<br>VSCKTSGY	TFTNFYIH	WVRQAPGQGL<br>EWMGV	INPNGGGTIYPQ<br>NFQG	RITMMSDTSTTSTSTVYM<br>ELSSLRSEDTAVYYCAR	GGKSCSGGSC<br>RYGPITLDF	WGQGT
**V_L**	MAELTQPHSVSGSPG<br>KTVTISC	TGSSGSIANN<br>FVQ	WYQQRPGSAPT<br>TVIY	EDDKRPS	GVPDRFSGSIDSSSNSASL<br>TISGLRTEDEADYYC	QSYAGSNVV	FGGGT

**Heavy Chain constant region**	HHHHFVQDLGSTFLSTLVLLGL

S1.4 Nanoparticles tracking analysis

NTA measurements were performed with a NanoSight LM10-HS system, (NanoSight, Amesbury, United Kingdom). NPs were diluted 1:100 in PBS 1X and then the sample was injected in the sample chamber with sterile syringes (BD Discardit II, New Jersey, USA) until the liquid reached the tip of the nozzle. Five recording (60 sec) were performed at room temperature. NTA software provided high-resolution particle size distribution profiles and concentration measurements. Dilution factors were used to calculate particle concentration.

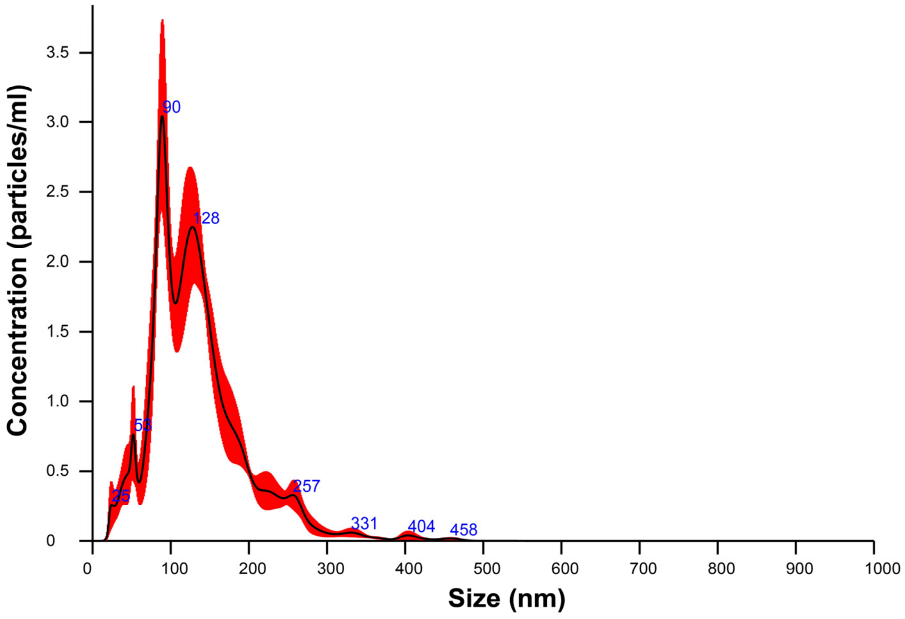

Figure S1. Nanoparticle tracking analysis on PLGA-PEG-Bis-Sulfone NPs

Table S4. SWOT analysis

Strenghts	Weaknesses	Opportunities	Threats
Accurate and highly sensitive diagnose	Inability to determine the concentration of Fab tethered to the NPs surface	Reduction of drug quantities via targeted *in situ* release	Legal complications for *in vivo* testing
High adaptability for different diagnostic and/or therapeutic approaches	Challenges in ensuring uniformity of Fab binding across NIPs	Identification of the infection site	High biotechnological competition
No cytotoxicity effects detected			
